# ARC^GHR^ Neurons Regulate Muscle Glucose Uptake

**DOI:** 10.3390/cells10051093

**Published:** 2021-05-03

**Authors:** Juliana Bezerra Medeiros de Lima, Lucas Kniess Debarba, Alan C. Rupp, Nathan Qi, Chidera Ubah, Manal Khan, Olesya Didyuk, Iven Ayyar, Madelynn Koch, Darleen A. Sandoval, Marianna Sadagurski

**Affiliations:** 1 Department of Biological Sciences, Integrative Biosciences Center (IBio), Wayne State University, Detroit, MI 48202, USA; lima@wayne.edu (J.B.M.d.L.); lucaskniess@wayne.edu (L.K.D.); gi9232@wayne.edu (C.U.); manal.khan@wayne.edu (M.K.); olesya.didyuk@wayne.edu (O.D.); iven.ayyar@wayne.edu (I.A.); madelynn.koch@wayne.edu (M.K.); 2 Division of Metabolism, Endocrinology and Diabetes, Department of Internal Medicine, University of Michigan Medical School, Ann Arbor, MI 48109, USA; ruppa@med.umich.edu (A.C.R.); nathanqi@med.umich.edu (N.Q.); 3 Department of Pediatrics, Anschutz Medical Campus, University of Colorado, Denver, CO 80217, USA; DARLEEN.SANDOVAL@cuanschutz.edu

**Keywords:** growth hormone receptor, arcuate nucleus of the hypothalamus, glucose metabolism, energy homeostasis

## Abstract

The growth hormone receptor (GHR) is expressed in brain regions that are known to participate in the regulation of energy homeostasis and glucose metabolism. We generated a novel transgenic mouse line (GHR^cre^) to characterize GHR-expressing neurons specifically in the arcuate nucleus of the hypothalamus (ARC). Here, we demonstrate that ARC^GHR+^ neurons are co-localized with agouti-related peptide (AgRP), growth hormone releasing hormone (GHRH), and somatostatin neurons, which are activated by GH stimulation. Using the designer receptors exclusively activated by designer drugs (DREADD) technique to control the ARC^GHR+^ neuronal activity, we demonstrate that the activation of ARC^GHR+^ neurons elevates a respiratory exchange ratio (RER) under both fed and fasted conditions. However, while the activation of ARC^GHR+^ promotes feeding, under fasting conditions, the activation of ARC^GHR+^ neurons promotes glucose over fat utilization in the body. This effect was accompanied by significant improvements in glucose tolerance, and was specific to GHR^+^ versus GHRH^+^ neurons. The activation of ARC^GHR+^ neurons increased glucose turnover and whole-body glycolysis, as revealed by hyperinsulinemic-euglycemic clamp studies. Remarkably, the increased insulin sensitivity upon the activation of ARC^GHR+^ neurons was tissue-specific, as the insulin-stimulated glucose uptake was specifically elevated in the skeletal muscle, in parallel with the increased expression of muscle glycolytic genes. Overall, our results identify the GHR-expressing neuronal population in the ARC as a major regulator of glycolysis and muscle insulin sensitivity in vivo.

## 1. Introduction

In the central nervous system (CNS), the growth hormone receptor (GHR) is present in regions known to participate in the regulation of feeding, energy balance, and glucose metabolism, including the hypothalamus, hippocampus, and amygdala [1,2,3]. The expression of GHR within the CNS has been mapped by in situ hybridization and through detection of the downstream target, phosphorylated signal transducer, and activator of transcription (STAT) 5, revealing large numbers of GH-responsive neurons in various brain regions [3]. While these studies detected GHR expression within the CNS, the functional assessment of GHR-expressing neurons in various brain regions is lacking. 

In recent years, it has become clear that GH action in the arcuate nucleus of the hypothalamus (ARC) represents an important component of energy homeostasis. We have recently shown that neuronal-specific deletion of GHR in leptin receptor (LepRb)-expressing neurons in the hypothalamus impaired hepatic glucose production and systemic lipid metabolism [4]. Additionally, mice lacking GHR, specifically in the orexigenic agouti-related peptide (AgRP) expressing neurons in the ARC, displayed impaired responses to fasting and food restriction, while the deletion of GHR from anorexigenic proopiomelanocortin (POMC) neurons in the ARC did not produce a significant metabolic phenotype [5,6]. Collectively, these results indicate unique roles of GHR signaling in the ARC neurons in metabolic control. However, it remains unknown whether GHR-expressing neurons in the ARC, per se, are directly involved in metabolic control.

In the current study, we specifically focused on studying the role of the in vivo activation of the GHR-expressing neurons in the ARC in the regulation of energy homeostasis and systemic glucose metabolism. To this end, we developed a novel GHR-driven cre mouse (GHR^cre^) that allowed us to both track and activate GHR-expressing neurons by directly enhancing the activity of the neurons in a non-invasive manner using designer receptors exclusively activated by designer drugs (DREADD) technology. We found that the activation of ARC^GHR+^ neurons increased the systemic glucose sensitivity, whole-body glycolysis, and insulin-stimulated muscle glucose uptake, accompanied with an elevation in muscle glycolytic genes. Overall, our study revealed a novel network of metabolic regulation through the hypothalamic GH responsive GHR axis in the ARC. 

## 2. Materials and Methods

### 2.1. Animals

GHR^cre^ mice were generated using the Clustered Regularly Interspaced Short Palindromic Repeats associated protein Cas9 (CRISPR/Cas9) at the University of Michigan Transgenic Core, as done previously [7]. A description of the procedures is described in the Supplementary Materials for *tdTomato* mice on the ROSA26 background (B6.Cg-Gt(ROSA)26Sort^m14(CAG-tdTomato)Hze^/J; #007914, Jackson Laboratory, Bar Harbor, ME, USA). The generation of growth hormone releasing hormone (Ghrh^cre^) mice was previously described [8]. To allow for the cre-dependent expression of stimulatory DREADD receptors (hM3DGq) from the ROSA26 locus, we crossed hM3DGq transgenic mice [9] with Ghrh^cre^ mice (GHRH::hM3DGq). Adult male mice (8–12 weeks old) were used for all of the studies. All procedures and experiments were carried out in accordance with the guidelines established by the National Institutes of Health Guide for the Care and Use of Laboratory Animals and approved by the University of Michigan and Wayne State University Institutional Animal Care and Use Committee. Approval number: IACUC-20-01-1712.

### 2.2. Surgery and Viral Injections

Stereotaxic viral injections were performed as described [10]. Briefly, animals were anesthetized using 1–3% isoflurane, and their heads were shaved and placed in a three-dimensional stereotaxic frame (Kopf 1900, Kopf instruments, Tujunga, CA, USA). This model provides a 1 micron precision and accuracy with a digital display readout. An alignment indicator provided a dimensional output with a 0.010 mm resolution to ensure head placement. The skull was exposed with a small incision, and two small holes were drilled for bilateral microinjection (200 nL/side) of the excitatory DREADD, AAV8-hSyn-DIO-hM3DGq-mCherry (#44361-AAV8, Addgene), into the ARC of the GHR^cre^ mice at stereotaxic coordinates based on the Mouse Brain Atlas, as follows: A/P: −1.3, M/L: +/−0.2, and D/V: −5.85 [11]. After surgery, the mice were given 2 weeks of recovery to maximize the virally-transduced gene expression and to acclimate the animals to handling and the experimental paradigms before the study. The activation of the DREADD receptor was induced by intraperitoneal administration of the agonist, clozapine-*n*-oxide (CNO, #4936, Tocris). The expression was verified post hoc in all animals, and any data from animals in which the transgene expression was located outside the targeted area were excluded from analysis. 

### 2.3. Metabolic Analysis

Following recovery, GHR^cre^ mice with activating DREADD (hM3Dq) underwent glucose metabolism and energy expenditure assays. Intraperitoneal glucose tolerance tests were performed on mice that were fasted for 6 h. The mice were administered 0.9% saline or CNO 1 h before glucose injection. Glucose tolerance tests (GTTs) were performed one week apart, and the blood glucose levels were measured as described before [12]. Blood insulin was determined using a Mouse Insulin ELISA kit (#50-194-7920, Crystal Chem. Inc., Elk Grove Village, IL, USA). For peripheral GH stimulation (recombinant mouse GH, 12.5 µg/100 g BW, Harbor-UCLA Medical Center, Torrance, CA, USA), the mice were injected i.p. and perfused 1.5 h later, as before [4]. Metabolic measurements of the energy homeostasis were obtained using an indirect calorimetry system (PhenoMaster, TSE system, Bad Homburg, Germany). The mice were acclimatized to the cages for 3 days. Following acclimatization, the mice were monitored for 5 days. In the morning of the first assessed day, the GHR-hM3Dq mice received an i.p. injection of the vehicle, and the measurements were analyzed for the following 8 h under fed or fasted conditions. The mice remained in the metabolic chambers with or without food and water *ad libitum*, and 72 h later, the same experimental design was repeated, but the animals were treated with an i.p. injection of CNO (0.3 mg/kg) instead. Fat oxidation was calculated with the formula 1.69VO_2_-1.69VCO_2_-2.03n, and glucose oxidation was calculated with the formula 4.57VCO_2_-3.23VO_2_-2.60n. Data were analyzed as vehicle vs. CNO per mouse. Chemogenetic activation of the GHRH neurons was achieved by injecting CNO 1 h prior to glucose administration. The experimental animals were hMD3Gq^lox/WT^ GHRH^Cre^ (GHRH::hM3DGq) and hMD3Gq^lox/WT^. 

### 2.4. Hyperinsulinemic-Euglycemic Clamp

At 16–18 weeks of age, GHR-hM3Dq mice underwent hyperinsulinemic-euglycemic clamp. The right jugular vein and carotid artery were surgically catheterized, and male mice were given 5 days to recover from the surgery. After 5–6 h of fasting, hyperinsulinemic-euglycemic clamp studies were performed on unrestrained, conscious mice using the protocol adopted from the Vanderbilt Mouse Metabolic Phenotyping Center [13] from the University of Michigan Animal Phenotyping Core, consisting of a 90-min equilibration period followed by a 120-min experimental period (t = 0–120 min). At 90 min prior to insulin infusion, a bolus infusion of 1 μCi of [3-3H]-glucose (HPLC purified, Perkin Elmer, Norwalk, CT, USA) was given, and was followed by a 0.05 μCi/min infusion that was increased to 0.1 μi/min during a 2-h clamp procedure to assess the basal and insulin-stimulated whole-body glucose turnover. Hyperinsulinemic-euglycemic clamp was initiated with a prime-continuous infusion (16 mU/kg bolus, followed by 4.0 mU/kg/min or 24 pmol/kg/min) of human insulin (Novo Nordisk, Bagsvaerd, Denmark). Euglycemia was maintained during the clamp by measuring the blood glucose sampled from an arterial catheter every 10 min, and by adjusting the infusion rate of 50% glucose accordingly. CNO was injected 1 h before the beginning of the clamp. The blood samples were collected during a steady-state of glucose infusion at t = 80, 85, 90, 100, 110, and 120 min for the determination of the glucose specific activity. The blood insulin concentrations were determined from the samples taken at t = −10 and 120 min. At the end of the experiment, animals were anesthetized with an intravenous infusion of sodium pentobarbital, and the tissues were collected and immediately frozen in liquid nitrogen for later analysis of the tissue 14C radioactivity. Plasma insulin was measured using millipore rat/mouse insulin ELISA kits. For determination of the plasma radioactivity of [3-3H]-glucose and 2-[1-14C] deoxyglucose, plasma samples were deproteinized and counted using a liquid scintillation counter. The plasma ^3^H_2_O in each sample was determined by the difference in the 3H counts between an aliquot of the sample that was dried to remove 3H_2_O, and another aliquot that was directly counted without a drying process. For the analysis of the tissue 2-[1-14C] deoxyglucose 6-phosphate, tissues were homogenized in 0.5% perchloric acid, and the supernatants were neutralized with KOH. Aliquots of the neutralized supernatant with and without deproteinization were counted for determination of the content of 2-[1-14C] deoxyglucose phosphate. An aliquot of the neutralized supernatant was directly counted for the total tissue counts of [^14^C] 2DG and [^14^C] 2DGP. Another aliquot was deproteinized with ZnSO4 and Ba(OH)2 to remove [^14^C]2DGP, and was counted for [^14^C]2DG only. Whole body glycolysis was estimated as appearance of ^3^H_2_O in the total body water, adjusted by the plasma specific activity of [3H] glucose, as before [14].

### 2.5. Perfusion and Histology

Mice were anesthetized (IP) with avertin and were transcardially perfused [15]. For the immunohistochemistry, free-floating brain sections were stained with the following primary antibodies: DsRed (anti-rabbit, 1:5000, #NC9580775, Takara, Ann Arbor, MI, USA), GFP (anti-chicken, 1:1000, #ab13970, Abcam, Cambridge, MA, USA), anti-tdTom (anti-goat, 1:500, #AB8181-200, Sicgen, Cantanhede, Portugal), pSTAT5 (anti-rabbit, 1:500, #9359, Cell Signaling, Danvers, MA, USA), glial fibrillary acidic protein (GFAP; anti-chicken, 1:500, #Ab5541, Millipore, Burlington, MA, USA), Iba-1 (anti-goat, 1:1000, #ab5076, Abcam, Cambridge, MA, USA), ß-Endorphin (anti-rabbit, 1:400, #H-022-33, Phoenix Pharm, Burlingame, CA, USA), tyrosine hydroxylase (anti-rabbit, 1:200, #AB152, Millipore, Burlington, MA, USA), and cFos (anti-sheep, 1:500, #ab6167, Abcam, Cambridge, MA, USA). For the staining specificity control, immunohistochemical experiments were performed with brain sections, in which the primary antibody was omitted and substituted with serum.

### 2.6. Two-Plex Fluorescent In Situ Hybridization

Fixed-frozen ARC-containing GHR^cre^ brain sections of 12-week old male mice (10 µm) were processed for the RNAscope Fluorescent Multiplex assay (Advanced Cell Diagnostics, Inc). The samples were double-labeled with probes for GHR (Mm-Ghr-C2 464951), GHRH (Mm-Ghrh-C2 470991), or somatostatin (SST; Mm-Sst-C2 404631), together with tdTom (*tdTomato*-C3 317041). Double-label *in situ* hybridization (ISH) and immunohistochemistry (IHC) were performed as previously described [16,17]. 

### 2.7. Images and Data Analysis

All of the sections used for ISH were blindly visualized with a Zeiss M2 microscope. All other fluorescent sections were visualized with a Nikon Eclipse Ni microscope coupled to a Nikon DS-Ri2 camera. The photomicrographs were captured using NIS-Elements Br 5.0 Zen software. Fiji ImageJ image-editing software was used to overlay the photomicrographs in order to construct merged images and to mount plates. Only the sharpness, contrast, and brightness were adjusted, and the same values for each target labeled were applied. 

### 2.8. RNA Extraction and qPCR

Gastrocnemius muscle samples were carefully dissected. RNA was isolated using the QIAGEN RNeasy Kit (QIAGEN, Valencia, CA, USA), which was combined with the RNase-Free DNase Set (QIAGEN, Valencia, CA, USA) and reverse-transcribed using the iScript cDNA kit (Bio-Rad Laboratories Inc., Hercules, CA, USA). Each reaction was carried out in triplicate, as previously described [12].

### 2.9. Statistical Analysis

Unless otherwise stated, mean values ± standard error of the mean (SEM) are presented in graphics. The GTT data were analyzed with a residual maximum likelihood (REML) mixed model followed by Sidak’s post hoc, while the energy homeostasis parameters were analyzed through repeated-measures two-way analysis of variance (ANOVA) followed by Sidak’s post hoc, from the time of vehicle or CNO injection to the end of light cycle. The same parameters were also measured as the average of 4 h after vehicle or CNO injection, and were analyzed as two-tailed paired *t*-test. Post-hoc comparisons were only carried out when the *p*-value was significant for the effect and/or interactions. *p* < 0.05 was considered statistically significant.

## 3. Results

### 3.1. Characterization of the GHR^cre^ Mice

**Figure 1:**
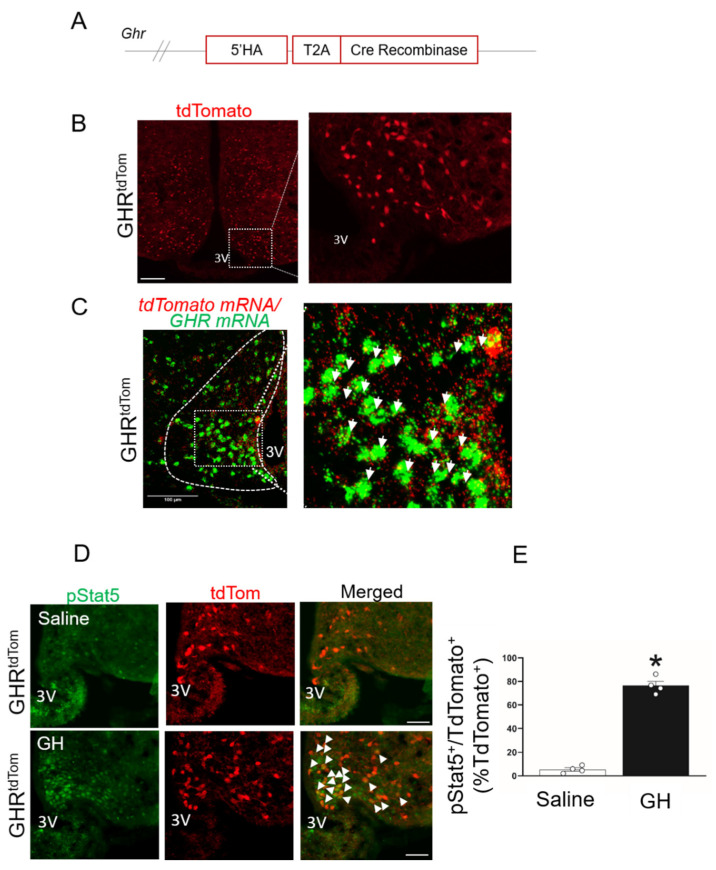
Characterization of growth hormone receptor (GHR)-expressing neurons in the arcuate nucleus of the hypothalamus (ARC). (**A**) Diagram of knock-in insert containing the cre enzyme. T2A—self-cleaving peptide; HA—homology arm. (**B**) Immunofluorescent image of GHR^+^ neurons in the hypothalamus (red, *TdTomato*). The dashed box indicates a digitally enlarged region of the ARC. (**C**) Two-plex fluorescent in situ hybridization of *GHR* mRNA (green) and *tdTomato* mRNA (red) in the ARC. The dashed box indicates the colocalization of *GHR* and *tdTomato* mRNA in the ARC (white arrows). (**D**) Representative images for pSTAT5 in 12-week-old GHR^tdTom^ mice injected i.p. with a vehicle (saline) or GH. pSTAT5 (green), *TdTomato* (red), and merged images (colocalization is shown by arrows). 3V—third ventricle. Scale bar: 100 µm. (**E**) Quantification of double labeled cells of pSTAT5 and *tdTomato* to the total % red-labeled cells.* *p* < 0.05 *t*-test. See also Figures S1–S3.

To characterize the role of GHR-expressing neurons in the ARC, we developed a GHR cell-specific molecular tool (GHR^cre^) using CRISPR/Cas9 gene-editing technology (Figure 1A). 

The GHR^cre^ mice were reproduced with the Mendelian ratio and exhibited a normal body weight and fed/fasting blood glucose levels (Supplementary Figures S1 and S2A). The GHR^cre^ mouse line was validated by a cre-dependent Rosa26-tdTomato reporter mouse. The expression pattern of the *tdTomato* reporter revealed the presence of GHR^cre^-expressing neurons in several areas of the hypothalamus (Figure 1B, Supplementary Figure S2B, and Supplementary Tables S1 and S2), including the midbrain and hindbrain. To validate the expression of GHR in our GHR^tdTom^ mice, we performed RNA in situ hybridization with RNAscope, using probes against *GHR* and *tdTomato* in the ARC. As seen in Figure 1C, the majority of *TdTomato*^+^ neurons were positive for the expression of the *GHR* gene. 

To track GH-mediated STAT5 phosphorylation (pSTAT5), an acute intraperitoneal GH injection was given to the GHR^tdTom^ mice. We found that pStat5 was colocalized with the majority of ARC^GHR+^ neurons in comparison with the saline-treated GHR^tdTom^ mice (*p* < 0.05, Figure 1D,E). We detected minimal colocalization of GHR^tdTom+^ cells in astrocytes positive to glial fibrillary acidic protein (GFAP; colocalization ~ 7%, Supplementary Figure S3A–C). Additionally, the Iba1, a marker of microglia, was co-localized with *tdTomato* at ~4%, (Supplementary Figure S3B,C), indicating that GHR signaling principally targets neurons and not glial cells. 

**Figure 2:**
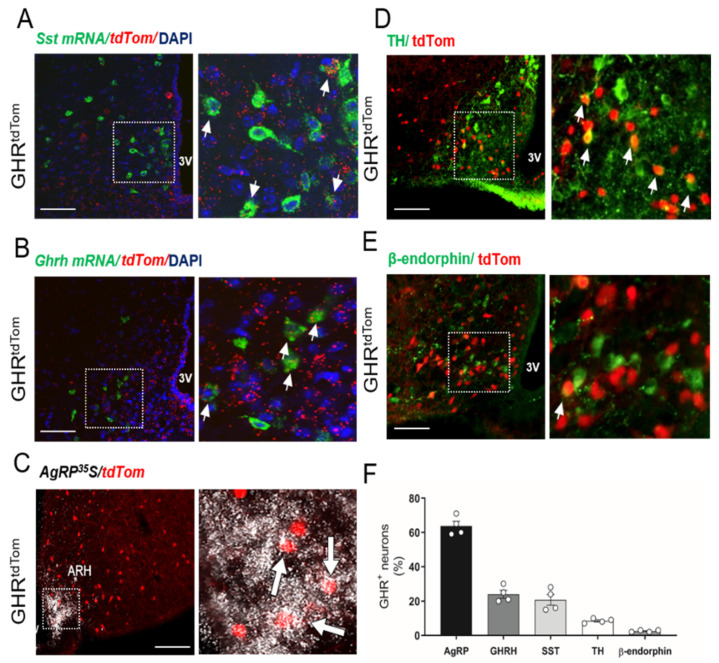
GHR-expressing neuron colocalization with ARC neuronal subpopulations. Two-plex fluorescent in situ hybridization of (**A**) *SST* mRNA (green), *tdTomato* mRNA (red), and *DAPI* (blue), and (**B**) growth hormone releasing hormone (*GHRH*) mRNA (green), *tdTomato* mRNA (red), and *DAPI* (blue). In situ hybridization of (**C**) *AgRP* mRNA (white) with immunohistochemistry for *tdTomato* (red). Immunofluorescent staining for (**D**) tyrosine hydroxylase (green) and (**E**) ß-endorphin (green) with *tdTomato* (red). The dashed box indicates digitally enlarged images demonstrating colocalization (white arrows). (**F**) Percentage of cells expressing targets in relation to the total amount of *TdTomato*^+^ (n = 4). 3V—third ventricle. Scale bar: 100 µm.

To determine whether ARC^GHR+^ neurons overlap with other known ARC populations that are involved in neuroendocrine regulation, we further examined the expression of *SST* and *GHRH* in the identified ARC^GHR+^ neurons using two-plex fluorescent ISH. We found colocalization of *GHRH* or *SST* and *tdTomato* mRNAs in the ARC of GHR^tdTom^ mice (Figure 2A,B,F). In support of previous studies [5,18], we further confirmed a substantial overlap of GHR^+^ neurons in the ARC with AgRP-expressing neurons in the GHR^tdTom^ mice (Figure 2C,F). Additionally, in support of single-cell sequencing data [18], we found a low (<10%) colocalization with dopaminergic neurons (GHR^tdTom+^/TH^+^ cells), and with POMC neurons (GHR^tdTom+^/β-endorphin^+^ cells, ~2–3%) in the ARC (Figure 2D–F).

### 3.2. ARC^GHR+^ Neurons Regulate Energy Homeostasis

**Figure 3:**
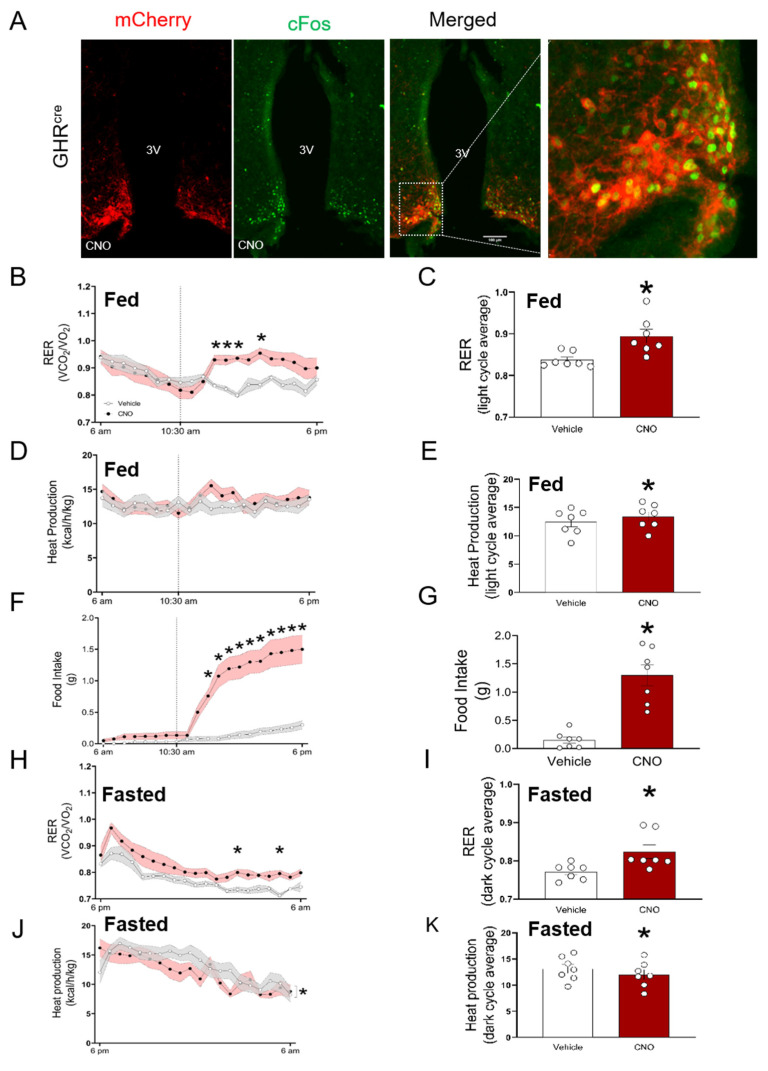
Acute activation of ARC^GHR^ neurons alters the energy homeostasis in fed and fasted mice. (**A**) Neuronal activation by cFos (green) was assessed 1 h after clozapine *n*-oxide (CNO) stimulation. immunohistochemistry (IHC) for mCherry (red) identifies the AAV-hM3Dq expression in the ARC^GHR+^ neurons. The merged image and dashed box indicate the colocalization of cFos and mCherry. 3V—third ventricle. Scale bar: 100 µm. Mice in metabolic chambers injected with saline (grey) or CNO (red) at 10:30 a.m. (**B**) Respiratory exchange ratio (RER) and (**C**) area under the curve (AUC) for the RER light cycle period. (**D**) Heat production and (**E**) AUC for heat production in the light cycle. (**F**) Food intake and (**G**) AUC of the light cycle period for food intake beginning at the treatment time. Mice in metabolic chambers were i.p. injected with either saline (grey) or CNO (red) at 5 pm, 1 h prior the start of the fasting period. (**H**) Respiratory exchange ratio (RER) and (**I**) AUC for RER dark cycle average. (**J**) Heat production and (**K**) AUC for heat production in the dark cycle. Data were analyzed by repeated measure two-way ANOVA followed by Tukey’s post-hoc; n = 7; * *p* < 0.05. See also Figure S5.

To establish the significance of ARC^GHR+^ neurons in the control of energy utilization, we employed a cre-dependent DREADD (designer receptors exclusively activated by designer drugs) virus to acutely modulate the neuronal activity in response to the peripheral injection of an otherwise inert compound, clozapine *n*-oxide (CNO) [10]. To determine whether the activation of ARC^GHR+^ neurons can influence energy homeostasis, we enhanced the ARC^GHR+^ neuronal activity of GHR^Cre^ mice by stereotactically injecting AAV8-DIO-hM3Dq-mCherry into the ARC (thereafter, GHR-hM3Dq mice), and activated the transduced cells with CNO. The activation of ARC^GHR+^ neurons is demonstrated by cFos expression as a marker of neuronal activation in GHR-hM3Dq mice treated with the vehicle or CNO (Figure 3A and Supplementary Figure S4A, *p* < 0.05). To analyze the components of energy expenditure, we injected ad libitum-fed 12-week-old male GHR-hM3Dq mice with CNO in the morning during the light cycle. Using a single-subject approach where each mouse served as its control, we showed that the stimulation of ARC^GHR+^ neurons produced a significant increase in the respiratory exchange ratio (RER) (Figure 3B,C *p* < 0.05). This effect was also associated with a slight increase in heat production. The total locomotor activity was not significantly different (Figure 3D,E and Supplementary Figure S5A,B). This effect was associated with an increase in food intake in these animals upon CNO administration (Figure 3F,G), suggesting that ARC^GHR+^ neurons are orexigenic neurons functionally similar to the AgRP/SST neuronal cluster in the ARC [18].

### 3.3. ARC^GHR+^ Neurons Regulate Glucose Tolerance

**Figure 4:**
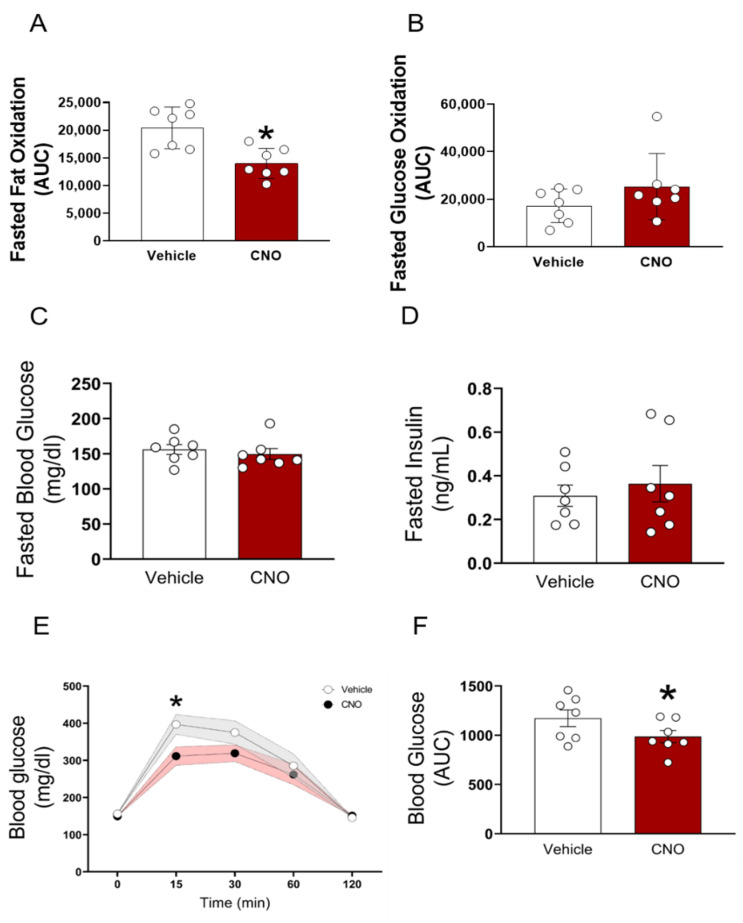
Acute activation of ARC^GHR^ neurons decreases fat oxidation and increases glucose tolerance. (**A**) Fasted fat oxidation and (**B**) fasted glucose oxidation, measured in metabolic chambers. Mice were i.p. injected with either saline (grey) or CNO (red) 1 h prior the start of the fasting period during the dark cycle. (**C**) Fasted blood glucose, (**D**) fasted insulin, (**E**) glucose tolerance tests (GTT), and (**F**) AUC of 12-week old male mice. Saline (gray) or CNO (0.3 mg/kg BW i.p, red) was injected 1 h before i.p. GTT. The effect of ARC^GHR^ activation was analyzed using a residual maximum likelihood (REML) mixed model followed by Sidak’s post hoc. The AUC was analyzed with a paired *t*-test. Mean ± SEM, n = 7; * *p* < 0.05. See also Figure S4B.

**Figure 5:**
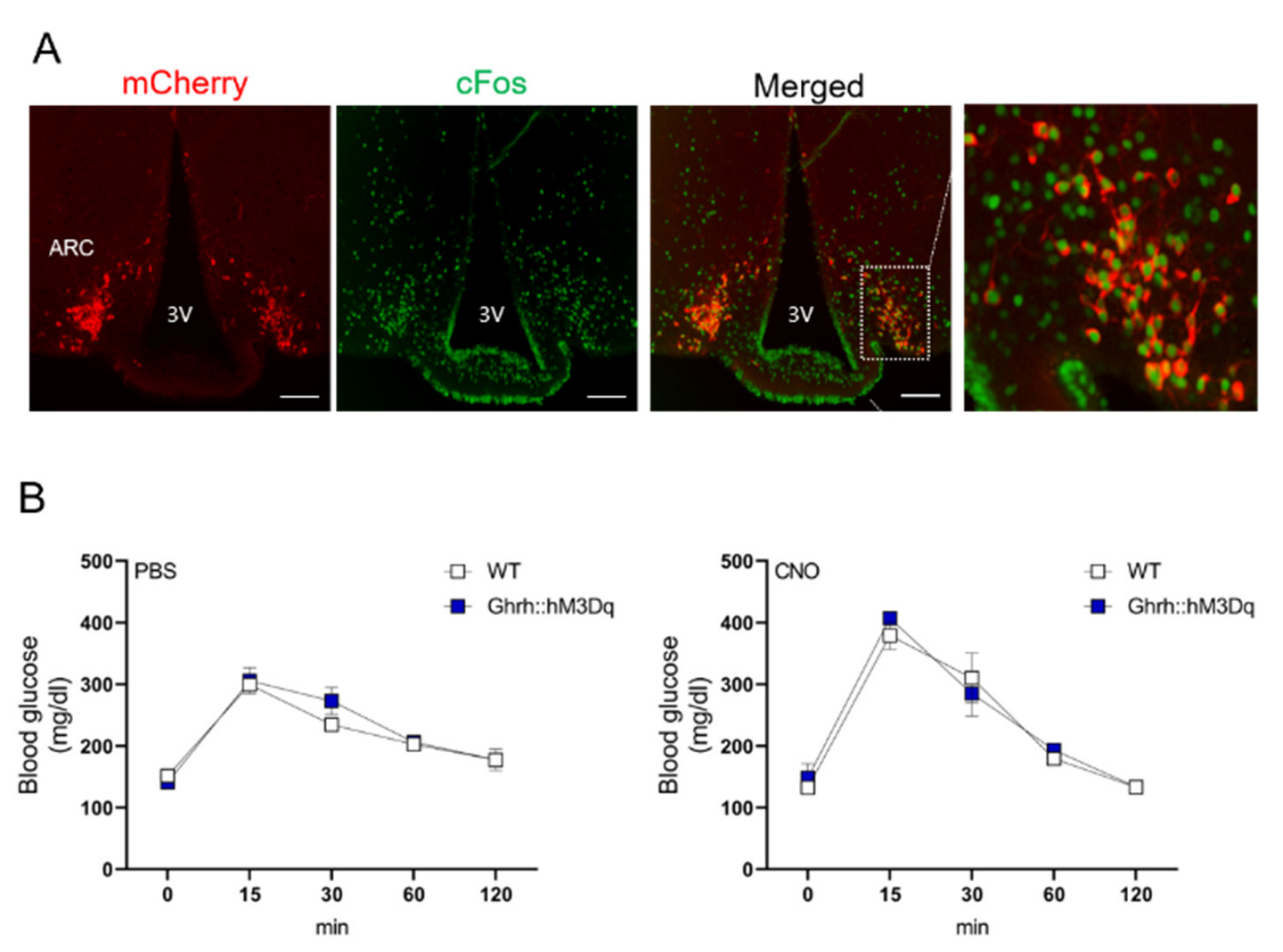
GHRH^+^ neuron activation does not alter metabolic parameters. (**A**) Representative immunofluorescence for Ghrh::hM3Dq mice injected with CNO. mCherry (red) and c-Fos (green), dashed box indicates the region of the ARC demonstrating colocalization. 3V—third ventricle. Scale bar: 100 µm. (**B**) Glucose tolerance tests (GTT) of 12-week old male control and Ghrh::hM3Dq mice performed one week apart. PBS or CNO was injected 1 h before i.p. glucose injection (n = 3).

The fasting blood glucose and serum insulin concentrations were indistinguishable between the vehicle and CNO-administered GHR-hM3Dq mice (Figure 4C,D). Despite unchanged fasting blood glucose levels, the CNO-treated mice displayed significantly increased glucose tolerance, indicating increased sensitivity in response to an intraperitoneal glucose load (*p* < 0.05, Figure 4E,F). Each animal served as its own control (e.g., saline vs. CNO). Of note, the hM3Dq virus alone injected into the control animals (hM3Dq_WT_) did not affect the glucose tolerance (Supplementary Figure S4B). To distinguish between different neuronal subsets within the GH axis, we next assessed the role of GHRH^+^ neurons in glucose tolerance. For this purpose, we utilized the recently generated GHRH^Cre^ mice. The expression pattern of GHRH in these animals demonstrated the presence of cre-expressing neurons in the expected areas of the hypothalamus, including the ARC [8]. For the cre-dependent expression of hM3DGq in the GHRH neurons, we crossed GHRH^cre^ mice with the stimulatory hM3DGq transgenic mice (GHRH::hM3Dq). CNO administration in GHRH::hM3Dq transgenic mice induced cFos immunoreactivity in the ARC of these mice (Figure 5A). In contrast to the ARC^GHR^ activation in the GHR-hM3Dq mice, the selective activation of all GHRH neurons in the GHRH::hM3Dq mice had no effect on the glucose tolerance (Figure 5B).

To circumvent the confounding effect of the altered food intake, we analyzed the components of energy expenditure in fasted animals. We activated GHR-hM3Dq mice with CNO, 1 h before the onset of fasting at the beginning of the dark cycle. Using the same approach, we show that during fasting, the activation of ARC^GHR+^ neurons by CNO produced a significant increase in RER compared with the vehicle (Figure 3H,I, *p* < 0.05). The heat production and locomotor activity were decreased (Figure 3J,K and Supplementary Figure S5C,D). Interestingly, fat oxidation was significantly decreased (*p* < 0.05), and there was a tendency for elevated glucose oxidation during fasting (Figure 4A,B). These data suggest that under fasting conditions, the activation of ARC^GHR+^ neurons promotes the utilization of glucose over fat in the body.

### 3.4. ARC^GHR+^ Neurons Control Whole-Body Glycolysis and Muscle Glucose Uptake

**Figure 6:**
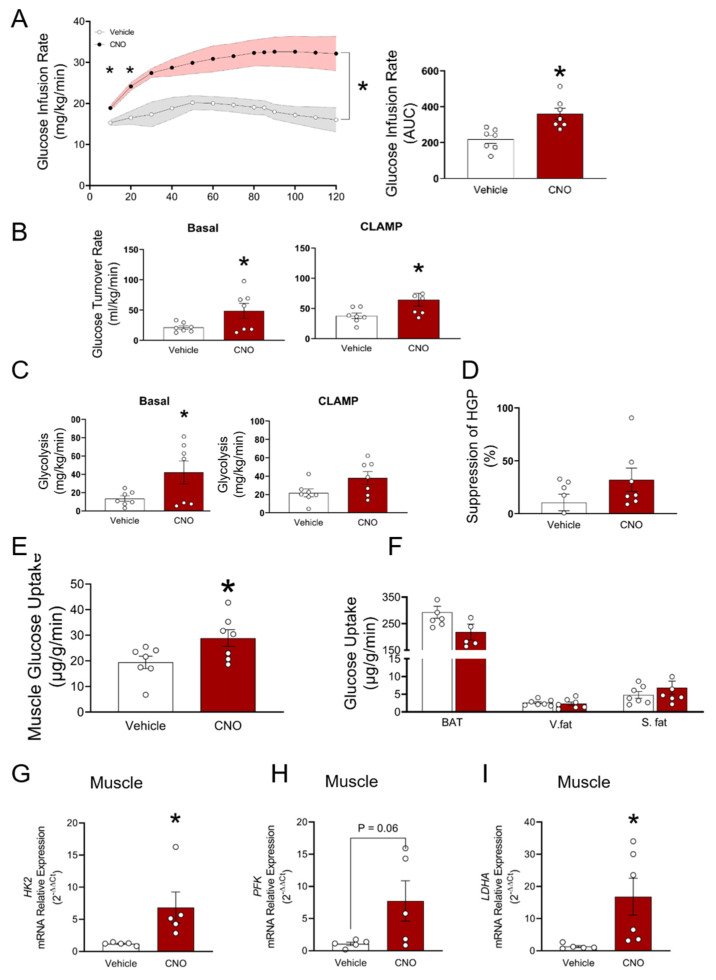
Hyperinsulinemic-euglycemic clamp during acute activation of ARC^GHR^ neurons. (**A**) Glucose infusion rates (GIR) and AUC. (**B**) Glucose turnover rate under basal and steady-state conditions. (**C**) Whole body glycolysis under basal and steady-state conditions. (**D**) Percentage suppression of hepatic glucose production. (**E**) Glucose uptake rates in skeletal muscle (gastroc, gastrocnemius muscle), and (**F**) brown adipose tissue (BAT), and white adipose tissue. V. fat—visceral fat; S. fat—subcutaneous fat. (**G**) *HkII,* (**H**) *Pfk,* and (**I**) *Ldha* mRNA expression in muscle in vehicle and CNO injected mice at 18–20 weeks of age; n = 7 mice per group. Mean ± SEM, *, *p* < 0.05.

We next performed a hyperinsulinemic-euglycemic clamp on GHR-hM3Dq mice. Consistent with an improvement in glucose tolerance, the glucose infusion rate (GIR) required to maintain euglycemia was nearly two-fold higher in the GHR-hM3Dq mice administered with CNO (AUC, *p* < 0.003, Figure 6A). Accordingly, the glucose turnover rate was elevated to a greater extent under basal and clamp conditions upon activation of the ARC^GHR+^ neurons (*p* < 0.004, Figure 6B). This was accompanied by a significant increase in the total body glycolytic rate (*p* < 0.05, Figure 6C), demonstrating that the activation of ARC^GHR+^ neurons stimulates glucose utilization. On the other hand, the suppression of hepatic glucose production (HGP) under clamp conditions was not significantly different between groups (Figure 6D). To identify the tissue(s) responsible for the increase in glucose utilization, we quantified the glucose uptake into insulin-responsive tissues. Skeletal muscle accounts for the majority of postprandial glucose disposal and, indeed, was significantly increased in the insulin-stimulated state in the GHR-hM3Dq mice upon CNO administration compared with the vehicle (*p* < 0.05, Figure 6E). Glucose utilization in the white adipose tissue (WAT) or brown adipose tissue (BAT) was not significantly different between groups (Figure 6F). In support of this, steady-state expression levels of the key metabolic genes that control glycolytic flux were greater in the muscle of CNO treated mice than in the control animals after the glucose clamp (Figure 6G–I). These data indicate that the observed increase in whole-body glucose tolerance upon activation of the ARC^GHR+^ neurons is driven mainly by the changes in glucose uptake by skeletal muscle.

## 4. Discussion

Our data identify a novel role for hypothalamic GHR-expressing neurons in the ARC in the regulation of whole-body glucose metabolism. Using a new mouse model that expresses cre recombinase driven by the GHR promoter, in combination with DREADD-mediated activation of ARC^GHR+^ neurons, we show that the chemogenetic activation of these cells strongly enhances glucose handling, as manifested by significant increases in the rates of whole-body glucose tolerance and glycolysis, as well as a specific increase in skeletal muscle insulin sensitivity.

ARC^GHR^ neurons represent a heterogeneous population, which includes neurochemically-defined neurons that control specific physiologic functions. For example, the acute chemogenetic activation of AgRP neurons alters food intake and decreases energy expenditure [10]. Additionally, the activation of AgRP neurons acutely impairs systemic insulin sensitivity by inhibiting glucose uptake in brown adipose tissue [9]. However, while the majority of GHR^+^ neurons in the ARC co-localize with AgRP neurons, *GHR* represents only a very small cluster within the AgRP neuronal population [18]; therefore, it remains possible that other GHR^+^ neuronal populations in the ARC contribute to ARC^GHR^-mediated enhanced glycemia. The activation of ARC^GHR+^ neurons in the fed state is probably AgRP-neuron dependent and, specifically, contributes to the regulation of short-term feeding by AgRP neurons [10]. In contrast, during fasting, ARC^GHR+^ neurons positively regulate systemic glucose sensitivity, suggesting that different neurocircuits are involved in the control of feeding compared with those activated at times of limited food availability.

Our clamp results indicate that activating ARC^GHR+^ neurons was robust in the basal state and during the hyperinsulinemic clamp. These results suggest that activating ARC^GHR+^ neurons can promote insulin-independent effects on whole-body glucose metabolism, in addition to the effects involving changes in the insulin action. Numerous studies indicate that the brain can stimulate insulin-independent glucose disposal. For example, in streptozotocin-induced diabetes rats, central administration of leptin can reverse hyperglycemia, regardless of insulin deficiency [19]. Additionally, hypothalamic infusion of FGF19 to leptin-deficient ob/ob mice increased insulin-independent glucose disposal [20]. Similarly, in a rat model of diabetes, the intracerebroventricular (i.c.v) injection of FGF1 delayed the onset of β-cell dysfunction without an effect on glucose-induced insulin secretion or insulin sensitivity, suggesting that FGF1 can act in the brain to stimulate insulin-independent glucose clearance [21]. Interestingly, some metabolic effects of FGF1 can involve the suppression of excessive hypothalamic–pituitary–adrenal axis activity [22]. Although a detailed understanding of mechanisms mediating the effect of ARC^GHR+^ neurons on the systemic glucose sensitivity awaits further study, our data support the model of hypothalamic insulin-independent control of blood glucose levels.

The majority of ARC^GHR^ neurons in the ARC are pSTAT5 immunoreactive after GH treatment, confirming their sensitivity to GH. The activation of ARC^GHR^ neurons modulates aspects of both glucose and energy homeostasis, indicating that ARC^GHR^ neurons lie within glucoregulatory and energy balance neurocircuits. While our current studies do not indicate which specific neuronal subpopulations within ARC^GHR^ are responsible for controlling whole-body glycolysis, genetic deletion of GHR in AgRP neurons did not affect glucose metabolism or energy homeostasis [5], indicating that the role of GHR in AgRP^-^ populations in the ARC is to coordinate these responses. ARC^GHR^ neurons only partially overlap with SST and GHRH neurons, thus the contribution of these ARC neuronal populations to ARC^GHR^-mediated metabolic effects remains to be clarified.

In summary, using a novel GHR^tdTom^ mouse model, we demonstrate that GHR neurons in the ARC comprise a unique neuronal population capable of controlling glucose metabolism and muscle insulin sensitivity. While the significance of the ARC subpopulation of GH-responsive neurons in the control of certain aspects of energy balance and glucose regulation remains to be elucidated, our study emphasizes the role of GH neurocircuitry as an essential hypothalamic network in regulating metabolic functions, and identifies these neurons as a promising therapeutic target for insulin resistance.

## Data Availability

The datasets generated and/or analyzed during the current study are available from the corresponding authors on reasonable request.

## Supplementary Materials

The following are available online at https://www.mdpi.com/article/10.3390/cells10051093/s1, Figure S1: GHRcre mice have normal body weight and fed glucose, Figure S2. TdTomato reporter fluorescent protein is expressed in various brain regions in GHRtdTom mice, Figure S3. GHR neurons colocalization with glia cells, Figure S4. hM3DqWT animals do not respond to CNO treatment, Figure S5. Acute activation of ARCGHR neurons increases ambulatory activity, Table S1. Distribution of GHRcre/cre TDTom immunoreactive neurons, Table S2. Distribution of GHRcre/cre TDTom immunoreactive neurons.

## Abbreviations

CNS	central nervous system
GH	growth hormone
GHR	growth hormone receptor
STAT5	signal transducer and activator of transcription 5
ARC	arcuate nucleus of the hypothalamus
SST	somatostatin
GHRH	growth hormone releasing hormone
AgRP	agouti-related peptide
