# Supplementary material for: ARCGHR Neurons Regulate Muscle Glucose Uptake
Source: Cells. 2021 May 3;10(5):1093. doi: 10.3390/cells10051093 (PMC8147615; doi:10.3390/cells10051093)
Supplement: Supplementary file 1 [file cells-10-01093-s001.zip › cells-1187714-supplementary.pdf]

cells

MDPI

SUPPLEMENTARY MATERIAL

Supplementary Material and Methods

GHR^{cre} mouse line generation:

Guide RNAs (sgRNA) targeting the 3′ end of the GHR coding region were selected using the computer algorithm available at http://crispor.tefor.net. sgRNAs were commercially synthesized by Synthego and tested in blastocysts to confirm DNA cutting. DNA was prepared from injected blastocysts and PCR amplification of the DNA at the targeted site was used to confirm DNA cutting. The editing template containing 1kb 5′-homology sequence, a T2A-Cre transgene replacing the stop codon and 1.5kb of 3′- homology was generated using Gibson Assembly. CRISPR reagents (sgRNA/Cas9/editing template) were then injected into 300 fertilized mouse eggs and transferred eggs to pseudo pregnant recipients to obtain pups. Tail DNA from 2-week old pups was then used to identify genetically modified mice. Following identification of GHR^{Cre} founders, quantitative PCR of genomic Cre was performed to determine the integration events in heterozygous and homozygous mice, and confirmed by DNA sequencing analysis. To validate the GHR^{Cre} mouse line, we crossed the founder mice (G0 generation) with wild type mice to expand the colony, and the Cre^{+} offspring (G1 generation) were bred to reporter mice that express tdTomato in a Cre-dependent manner. Cre-induced tdTomato was assessed in a series of brain sections from GHR^{Cre}-tdTomato reporter mice in G2 and subsequent generations.

Primers List:

Gene	Forward	Reverse
18S	GTAACCCGTTGAACCCCATT	CCATCCAATCGGTAGTAGCG
HK2	TGTCCCTACCTTTGGGTTTC	ATGCCTTGAATCCCTTTGTC
PFK	GGCCAATCCTCAAAATCCTA	CCAGACCGTTTCCTTGAAAT
LDHA	TGTGTGGAGTGGTGTGAATG	ACCTGCTTGTGAACCTCCTT

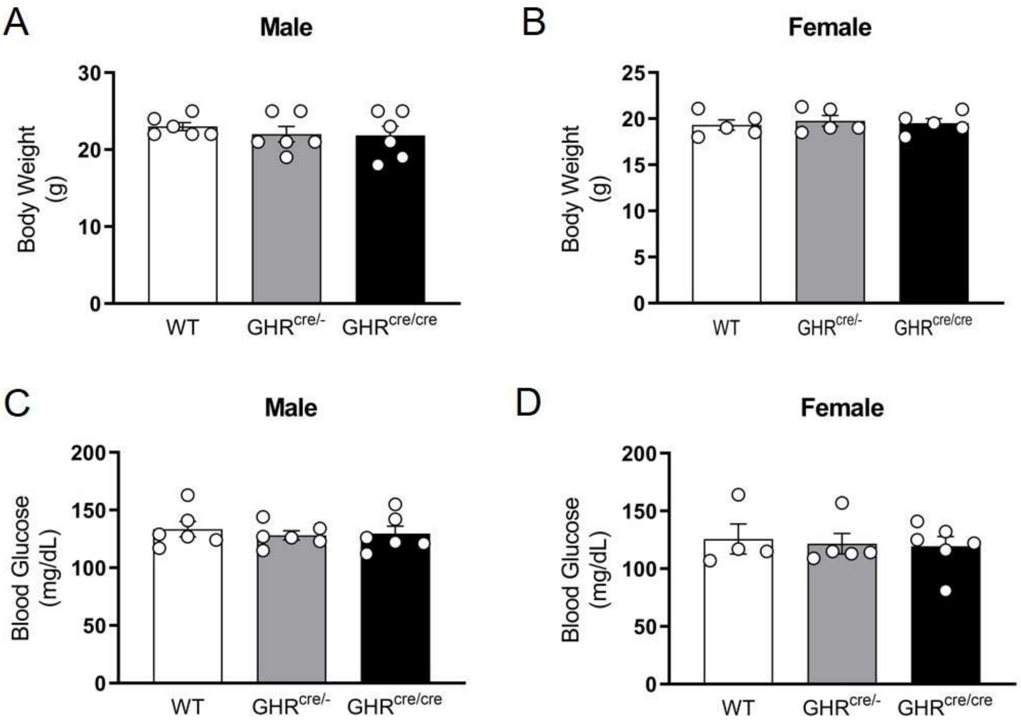

Figure S1. GHR^{cre} mice have normal body weight and fed glucose. Male (A) and female (B) body weight of wild-type (white bars), GHR$^{cre/-}$ (grey bars) and GHR$^{cre/cre}$ mice (black bars). Male (C) and female (D) fed blood glucose of wild-type

(white bars), $GHR^{cre/-}$ (grey bars) and $GHR^{cre/cre}$ mice (black bars). Results are presented as mean ± SEM and were analyzed through one-way analysis of variance (ANOVA).

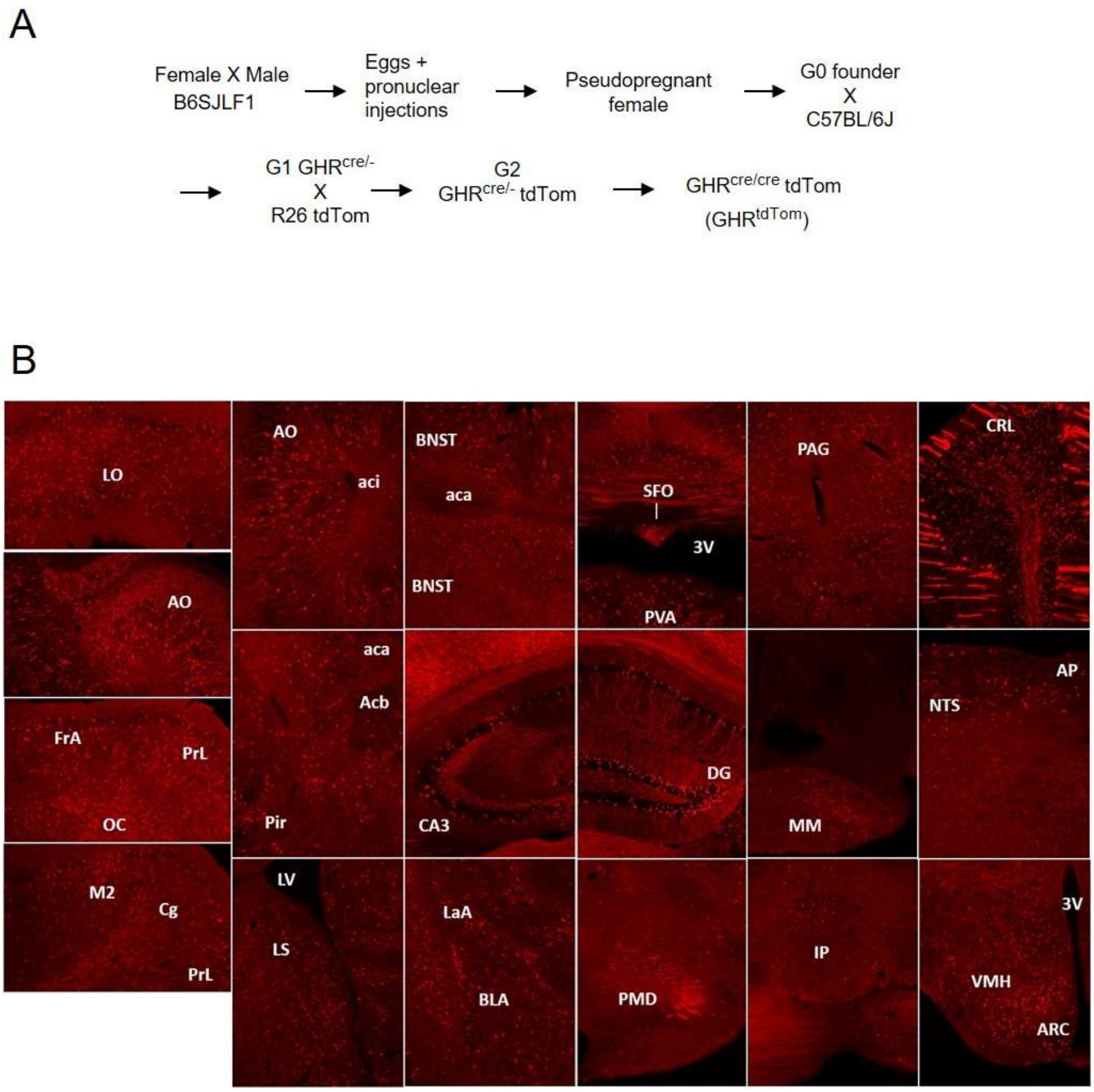

Figure S2. TdTomato reporter fluorescent protein is expressed in various brain regions in GHR^{tdTom} mice. (A) Breeding scheme for the generation of GHR^{tdTom} mouse line from the crossing of knock-in GHR^{cre} mouse with the reporter mouse. (B) Qualitative photomicrographs of brain regions expressing tdTomato reporter fluorescent (red). LO = Lateral orbital cortex, AO = Anterior olfactory area, OC = Orbital cortex, FrA Frontal association cortex, PrL = Prelimbic cortex, M2 = Secondary motor cortex, Cg = Cingulate cortex, aci = anterior commissure, aca = anterior portion of anterior commissure, Acb = Accumbens nucleus, Pir = Piriform cortex, LV = Lateral ventricle, LS = Lateral septal nucleus, BNST = Bed nucleus of the stria terminalis, CA3 = Field CA3 hippocampus, LaA = Lateral amygdala, BLA = Basolateral amygdala, SFO = subfornical organ, 3V = Third ventricle, PVA = Paraventricular thalamic nucleus, DG = Dentate gyrus, PMD = Dorsal premammillary nucleus, PAG = Periaqueductal gray, MM = Mammillary nucleus, IP = Interpeduncular nucleus, CRL = Cerebellum, NTS = Nucleus of solitary tract, AP = Area postrema. 3V = Third ventricle. Scale bar: 100 µm.

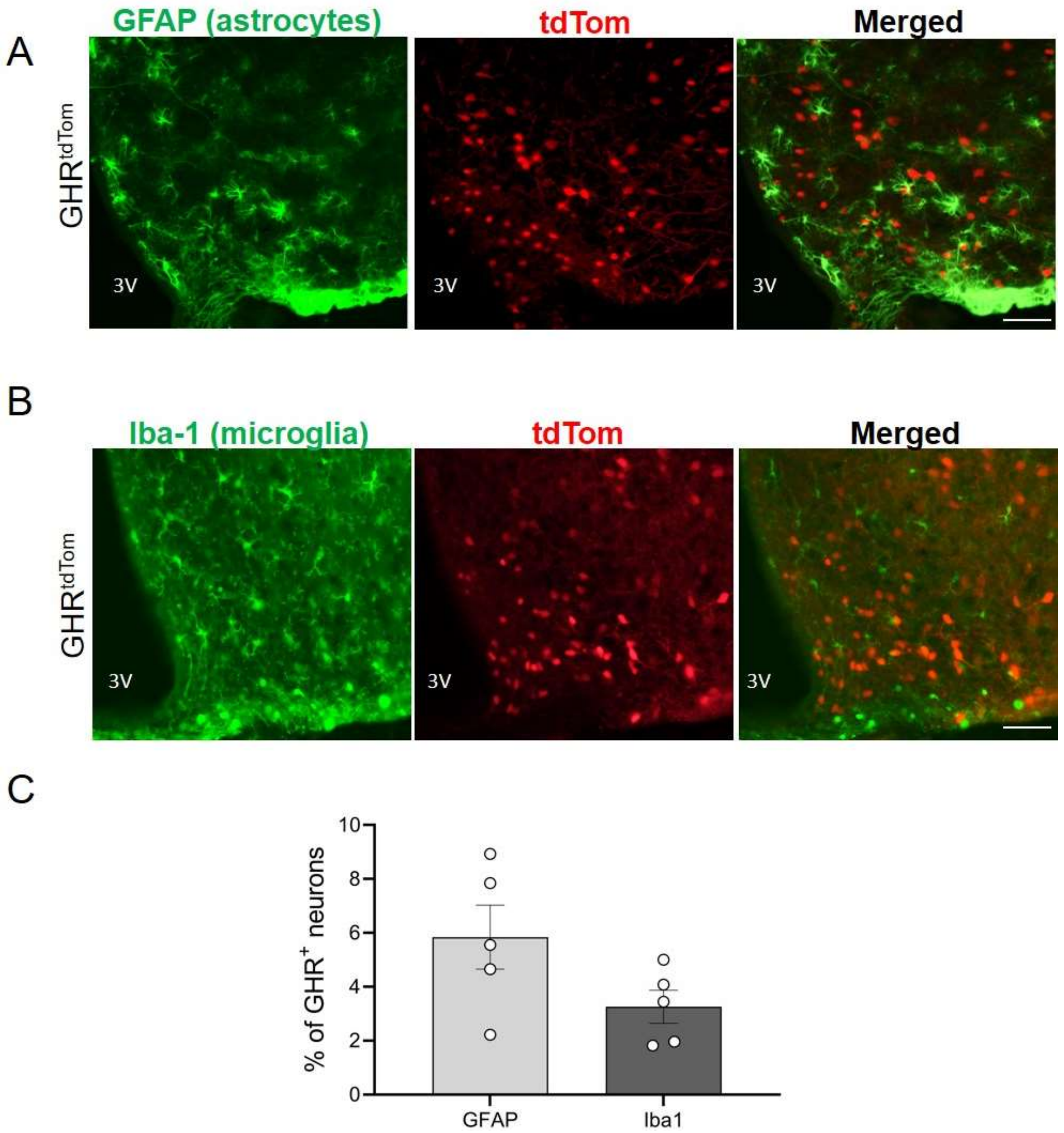

Figure S3. GHR neurons colocalization with glia cells. (A) Representative images of astrocytes identified by immunofluorescent detection of GFAP protein (green, upper panel) and (B) microglia evaluated by Iba1 immunostaining (green, lower panel) in the ARC obtained from GHR^{tdTom} mice (red, TdTomato). (C) Percentage of GHR^{+} neurons (n=5). 3V = Third ventricle. Scale bar: 100 µm.

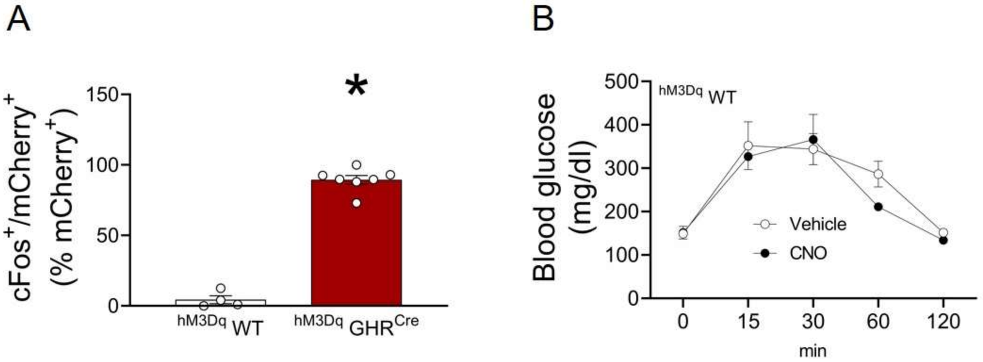

Figure S4. $hM3Dq^{WT}$ animals do not respond to CNO treatment. (A) Percentage of ARC c-Fos immunoreactive neurons 1 hour after CNO (0.3 mg/kg BW i.p) in WT (white bar) and $hM3DqGHR^{cre}$ (red bar) in relation to the total of tdTomato-positive cells. *$p < 0.05$ two-tailed t-test (n=4). (B) Glucose tolerance test (GTT) of $hM3Dq^{WT}$ 12-week-old male mice performed one week apart. Saline (0.1 mL/10 g BW – vehicle) or CNO was injected 1 hour before i.p. glucose injection (n=4).

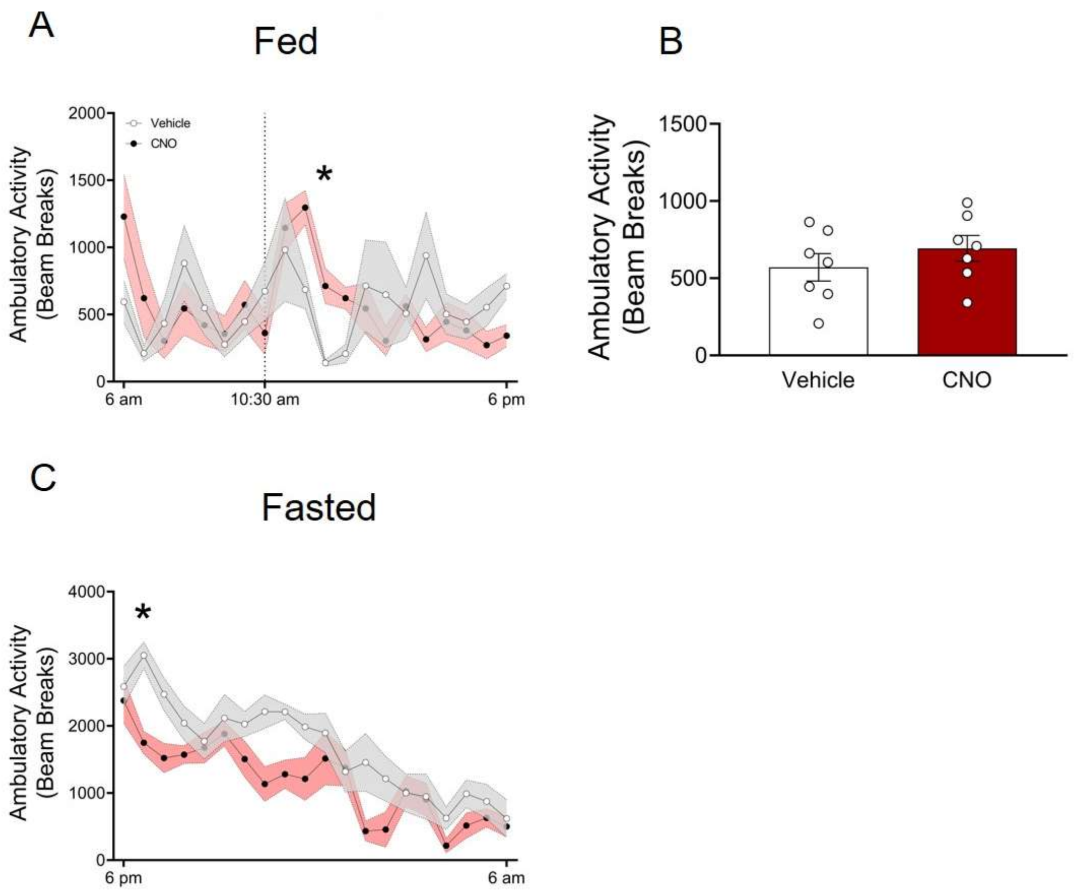

Figure S5. Acute activation of ARC^{GHR} neurons increases ambulatory activity. Mice were acclimated in metabolic cages and i.p. injected with either saline (grey) or CNO (red) at 10:30 am. (A) Fed ambulatory activity assessed by a total of beam breaks. (B) AUC of the light cycle period from the treatment time. (C) Fasted ambulatory activity assessed by a total of beam breaks (from 6 pm-6 am). (D) AUC of the dark cycle period from the CNO treatment time. Data are from male mice, analyzed by paired t-test (mean ± SEM, n = 7; * $p < 0.05$ compared to vehicle values).

Table 1. Distribution of GHR$^{cre/cre}$ TDTom immunoreactive neurons.

	BRAIN AREAS AND NUCLEI	GHR$^{cre/cre}$ tdTom-ir
LO	Lateral orbital cortex	++++
AO	Anterior olfactory area	++++
OC	Orbital cortex	++
PRL	Prelimbic cortex	+++
M2	Secondary motor cortex	++
CG	Cingulate cortex	++
PIR	Piriform cortex	++
ACB	Accumbens nucleus	++
LS	Lateral septal nucleus	+++
BNST	Bed nucleus of the stria terminalis	++
SFO	Subfornical organ	++++
PVA	Paraventricular thalamic nucleus	+++
DG	Dentate gyrus	+++
CA3	Field CA3 hippocampus	++
LAA	Lateral amygdala	++
BLA	Basolateral amygdala	++
PMD	Dorsal premammillary nucleus	+

**PAG**	Periaqueductal gray	++
**MM**	Mammillary nucleus	++
**IP**	Interpeduncular nucleus	+
**CRL**	Cerebellum	++++
**NTS**	Nucleus of solitary tract	++
**AP**	Area postrema	++

Table S2. Distribution of GHR$^{cre/cre}$ TDTom immunoreactive neurons.

**HYPOTHALAMIC AREAS AND NUCLEI**		**GHR$^{cre/cre}$ tdTom-ir**
**MPOA**	Medial preoptic area	+
**RCH**	Retrochiasmatic area	++
**PVH**	Paraventricular nucleus	++
**ARH**	Arcuate nucleus	++
**VMH**	Ventromedial nucleus	+++
**DMH**	Dorsomedial nucleus	+++
**LHA**	Lateral hypothalamic area	+
**DA**	Dorsal hypothalamic area	+
**PHA**	Posterior hypothalamic area	+++
**PMV**	Ventral premammillary nucleus	++
